# First-Line Treatment with Tivozanib for Metastatic Renal Cell Carcinoma in Real-World Settings Across Germany: Results of the Prospective, Non-Interventional, Post-Approval Study T-Rex

**DOI:** 10.3390/cancers17243910

**Published:** 2025-12-07

**Authors:** Viktor Grünwald, Karen Rußwurm, Ralf Eckert, Sandra Seseke, Diana Standhaft, Miriam Hegemann, Steffen Baumann, Horst Brenneis, Michael Seidel, Olrik Rau, Silke Schirrmacher-Memmel, Eva Hellmis, Claus F. Fieseler, Christian Doehn, Carsten Ziske, Andrea Distelrath, Norbert Marschner, Philipp Ivanyi, Martin Herold, Bianca I. Loehr, Carsten Lange, Andreas Janitzky, Martin Bögemann

**Affiliations:** 1 Institute for Urologic Oncology, Essen University Hospital, 45147 Essen, Germany; 2 Hematology, Oncology & Immunology, Bonifatius Hospital Lingen, 49808 Lingen, Germany; 3 Urologicum Eisleben, 06295 Eisleben, Germany; 4 Urological Practice, 06108 Halle, Germany; 5 Clinic for Urology, Hospital Dessau, 06846 Dessau, Germany; 6 Urological Clinic Sindelfingen, 71065 Sindelfingen, Germany; 7 Urological Practice, 04129 Leipzig, Germany; 8 Urological Practice, 66953 Pirmasens, Germany; 9 Group Practice for Urology, 04315 Leipzig, Germany; 10 Urological Practice, 38855 Wernigerode, Germany; 11 Oncological Practice, 63739 Aschaffenburg, Germany; 12 Urologicum Duisburg, 47169 Duisburg, Germany; 13 Urological Practice, 88048 Friedrichshafen, Germany; 14 Urologikum Lübeck, 23566 Lübeck, Germany; 15 GFO Kliniken, 53840 Troisdorf, Germany; 16 MVZ for Oncology and Urology, 26389 Wilhelmshaven, Germany; 17 Practice for Interdisciplinary Oncology and Hematology, 79110 Freiburg, Germany; 18 Hannover Medical School, 30625 Hannover, Germany; 19 Recordati Rare Diseases Germany GmbH, 89075 Ulm, Germany; 20 Urological-Oncology Practice, 06406 Bernburg, Germany; 21 Clinic for Urology, University Hospital Magdeburg, 39120 Magdeburg, Germany; 22 Urology and Pediatric Urology, University Hospital Münster, 48149 Münster, Germany

**Keywords:** advanced and metastatic renal cell carcinoma, tivozanib, tyrosine kinase inhibitor, patient-reported outcomes (PRO), Patient-Reported Outcomes version of the Common Terminology Criteria for Adverse Events (PRO-CTCAE)

## Abstract

Background: The efficacy and safety of tivozanib for the treatment of advanced or metastatic renal cell carcinoma (mRCC) have been established in the first-line setting in the Phase III trial TIVO-1. Methods: The prospective T-Rex study conducted in German clinical practice evaluated the safety, effectiveness and impact on quality of life (QoL) of first-line treatment with tivozanib in 32 patients with mRCC recruited between May 2019 and April 2021. Results: Recruited patients were predominantly elderly, with 53.1% aged over 75 years. Patients received a median of 6.5 tivozanib treatment cycles and the median time on treatment was 5.7 months. Overall, 78.1% of patients experienced treatment-related adverse events, including diarrhea, nausea and hypotension/hypertension. A clinical (i.e., complete or partial) response was observed in 46.9% of patients. Patients’ QoL remained stable from baseline to the end of treatment and most symptomatic toxicities resolved by the final treatment cycle, with the exclusion of dry skin, itching, and hand–foot syndrome. Conclusions: These data demonstrate that first-line treatment with tivozanib was associated with clinical activity, favorable tolerability, and stable QoL in patients with mRCC treated in everyday clinical practice across Germany, including those with advanced age.

## Simple Summary

Tivozanib is a tyrosine kinase inhibitor approved for the treatment of metastatic renal cell carcinoma in Europe based on the results of a Phase III trial. To complement the data from the clinical trial, we conducted the post-approval study T-Rex to assess tivozanib’s safety, effectiveness and impact on the patients’ quality of life when used to treat patients with metastatic renal cell carcinoma in routine clinical practice in Germany. The majority of the patients were over 75 years of age and did not previously receive treatment for renal cell carcinoma. Tivozanib was generally well tolerated and effective, with 46.9% of patients responding to treatment. Further benefits were reflected in the trend toward improved patient quality of life over the course of the study. Our findings support the use of tivozanib for the first-line treatment of patients with metastatic renal cell carcinoma, including those with advanced age, in routine clinical practice.

## 1. Introduction

Renal cell carcinoma (RCC) is the most common type of kidney cancer, accounting for ~90% of all kidney tumors, and ~3% of all cancers globally [1,2]. Clear cell RCC constitutes 80% of malignant renal tumors in adults, with papillary RCCs representing the majority of the remaining 20% of RCC cases [1]. The incidence of RCC typically peaks between the ages of 60 and 70 years and most patients are diagnosed with RCC over the age of 65 years [3]. Approximately 16% of patients with RCC present with advanced or metastatic renal cell carcinoma (mRCC) at diagnosis, and up to 20% of those initially diagnosed with localized RCC will develop metastases over the course of their disease [4,5].

Prior to the introduction of targeted therapies for mRCC, patients were treated with high-dose interleukin-2 or interferon-α, achieving median survival rates of <20 months [6,7]. The shift from these non-targeted therapies to targeted treatments, such as tyrosine kinase inhibitors (TKIs), immune checkpoint inhibitors (ICIs), and mammalian target of rapamycin (mTOR) inhibitors, has resulted in improved survival rates [8,9,10,11]. For example, in the COMPARZ and TIVO-1 studies, treatment with TKI monotherapy (pazopanib or sunitinib and sorafenib or tivozanib, respectively) demonstrated median overall survival rates of >28 months [9,10]. Overall survival improved even further in the era of ICI combination therapies [12,13,14,15].

Treatment decisions in patients with mRCC are guided by the stratification of patients into risk groups (favorable-, intermediate-, and poor-risk) proposed by the International Metastatic Renal Cell Carcinoma Database Consortium (IMDC) [1,16]. Recent evidence suggests that patients classified as favorable-risk (0 risk factors) have a higher proportion of angiogenic tumors and thus, are more sensitive to vascular endothelial growth factor (VEGF)-targeted therapies, compared with those in the intermediate- or poor-risk categories, [17,18,19]. VEGF plays a key role in tumor angiogenesis and vascular permeability, with VEGF overproduction shown to promote disease progression in patients with mRCC [4,20,21,22].

Combination therapy with ICIs and VEGF receptor-TKI is recommended as a first-line therapy in patients with clear cell RCC regardless of IMDC risk group [1,23]. However, monotherapy with TKIs, such as tivozanib, is recommended as an alternative to combination therapy in the IMDC favorable-risk group, or when ICI therapy is contraindicated [1,23]. While Phase III trials demonstrated robust survival benefits for regimens combining TKIs with ICIs in the overall patient population, subgroups of patients with intermediate- and poor-risk prognosis derived the most benefit, with mixed results in the favorable-risk subgroup [12,13,24,25,26]. These data suggest that the favorable-risk category is enriched for patients with angiogenic tumors who derive a greater survival advantage from VEGF-targeted therapies compared with those in the intermediate- and poor-risk categories [16]. Observations regarding the survival benefit of ICI combinations and TKI monotherapy across IMDC subgroups are reflected in the European Association of Urology (EAU) and European Society for Medical Oncology (ESMO) guidelines, which recommend TKI monotherapy for favorable-risk patients as an alternative to ICI combinations, and for all risk groups when ICI treatments are contraindicated [1,23].

One of the VEGFR-TKIs recommended for the treatment of mRCC is tivozanib, which is approved in Europe as first-line treatment for advanced RCC, and as second-line treatment in VEGFR and mTOR inhibitor-naïve patients with advanced RCC after disease progression following cytokine therapy [27]. Approval of tivozanib was based on the results of TIVO-1, a Phase III, multicenter, open-label, randomized study demonstrating superiority of tivozanib over sorafenib in both progression-free survival and objective response rate [10,27].

While clinical trials are important for evaluating the safety and efficacy of new therapies, they are subject to time limitations and strict eligibility criteria [28,29,30,31]. Post-approval studies can complement clinical trials by providing long-term data in a more diverse group of patients, thereby reducing selection bias and more accurately reflecting the target patient population seen in clinical practice [28,32,33]. Notably, while elderly patients are not always excluded from clinical trials based on age at enrollment [12,25,34,35,36], recruitment protocols may implicitly select for younger populations by restricting entry for individuals on the grounds of factors such as performance status, organ function or burden of study procedures [36,37,38]. In addition, post-approval studies sometimes evaluate patient-reported outcomes (PRO), which provide insight into the impact of a therapy from the perspective of the patient and can support clinical decision-making, facilitate communication between patients and healthcare professionals, and enable the monitoring of patient well-being and QoL in clinical practice [39,40,41].

Here, we report the results of the prospective, post-approval study T-Rex that evaluated the effectiveness, safety, and impact on QoL of tivozanib in patients with advanced or mRCC in routine clinical practice in Germany.

## 2. Materials and Methods

### 2.1. Study Design and Patient Population

The T-Rex study was a prospective, non-interventional, post-approval study of tivozanib in patients with advanced or mRCC. Recruitment commenced on 21 May 2019 and stopped early on 30 April 2021. During this period, patients diagnosed with advanced or mRCC were recruited in 23 centers across Germany, 21 of which had patients who received first-line treatment with tivozanib under clinical practice conditions and were eligible for this analysis. The database was closed on 15 November 2021. Eligible patients were aged ≥18 years with advanced or mRCC, with or without measurable disease according to RECIST 1.1 or based on clinical criteria at enrolment. The primary objective of the study was to evaluate the safety and effectiveness of tivozanib as well as its impact on QoL in patients with advanced or mRCC.

### 2.2. Study Endpoints and Assessments

The primary endpoints were the incidence of treatment-related adverse events and serious adverse events during the study and up to 30 days following the last dose of tivozanib, and PRO (QoL and tolerability to treatment). Secondary endpoints included tivozanib-related treatment discontinuations and hospitalizations, clinical response rate, duration of response, progression-free survival and duration of tivozanib treatment.

Tumor assessments were performed using imaging at intervals indicated by the treating physician and tumor response was evaluated according to the Response Evaluation Criteria In Solid Tumors (RECIST) Version 1.1, where available, or according to non-standardized clinical criteria. The clinical response rate was defined as the proportion of patients with complete and partial response. Progression-free survival was defined as the length of time from treatment initiation to disease progression or death and was estimated using the Kaplan–Meier method [42,43].

Safety assessments included the incidence of adverse events, rates of hospitalization and treatment discontinuation. Treatment-related adverse events and serious adverse events were monitored throughout the study and up to 30 days following the last dose of tivozanib and evaluated according to the Common Terminology Criteria for Adverse Events (CTCAE [V4.0]).

PRO included QoL and tolerability to treatment and were assessed using questionnaires, which were completed by all patients following every other tivozanib treatment cycle. QoL was assessed using the National Comprehensive Cancer Network Functional Assessment for Cancer Therapy—Kidney Symptom Index (19-item version; NCCN-FACT-FKSI-19 [Version 2]) questionnaire and the Patient-Reported Outcomes version of the Common Terminology Criteria for Adverse Events (PRO-CTCAE). The NCCN-FACT-FKSI-19 captures patient-reported data on QoL sub-scales over the previous seven days. In our study, the following four statements from the FKSI were chosen as they focus on emotional symptoms and functional well-being. Thus following patient responses were evaluated: ‘I am able to work (include work at home)’, ‘I am able to enjoy life’, ‘I am content with the quality of my life right now’, and ‘I worry that my condition will get worse’ [44]. For scores on the sub-scales ‘I am able to work (include work at home)’, ‘I am able to enjoy my life’, and ‘I am content with the quality of my life right now’ a higher score indicates improved patient QoL, whereas a higher score for the sub-scale ‘I worry that my condition will deteriorate’ suggests worsening of QoL. The PRO-CTCAE is a PRO measure that assesses up to 124 items relating to the attributes (frequency, severity, and interference with daily activities) of symptomatic toxicities experienced by patients over the previous seven days [45]. In the present study, a customized version with 61 items was evaluated. Attributes of reported toxicities were scored by patients from 1 to 5, with 5 indicating the absence of the symptom and lower scores indicating greater frequency, severity and interference with daily activities.

All statistical analyses were descriptive. Both the effectiveness and safety populations included all patients treated with tivozanib in the first-line setting.

## 3. Results

### 3.1. Patient Characteristics

**Figure 1:**
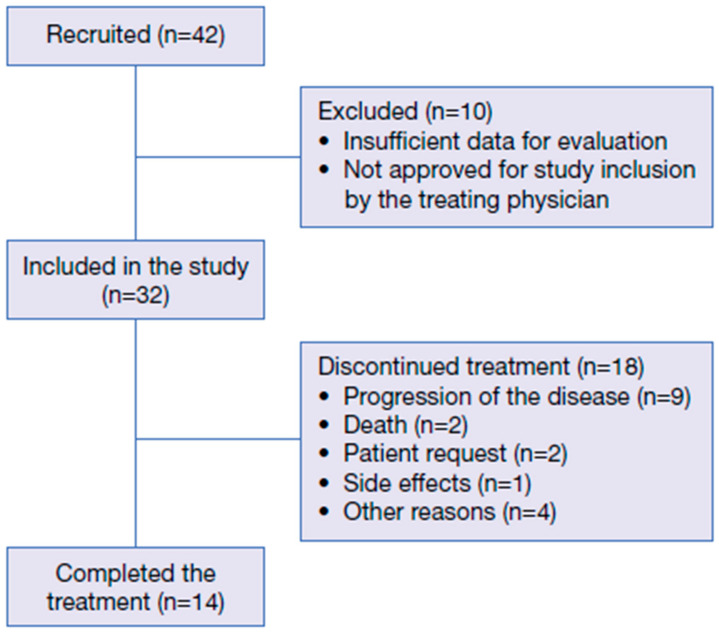
Patient disposition.

Overall, 42 patients were included in the study; however, ten patients did not have sufficient data for evaluation and/or were not approved for study inclusion by the treating physician (Figure 1).

Baseline patient and treatment characteristics of all 32 evaluated patients are shown in Table 1. The majority of patients were male (59.4%) and the median age was 77.5 years (range 61.0–89.0), with six (18.8%), nine (28.1%) and 17 (53.1%) patients aged ≤65 years, 66–75 years and >75 years, respectively. Twenty-one patients (65.6%) had a favorable European Cooperative Oncology Group Performance Status (ECOG PS) of 0, nine (28.1%) had a score of 1 and two (6.3%) had a score of 2. The majority of patients (n = 22, 68.8%) had an intermediate IMDC risk score, seven (21.8%) had a favorable and three (9.4%) a poor risk score. The mean Charlson Comorbidity Index (CCI), when excluding index points given for age and the presence of solid tumors, was 3.5 (SD 3.36) (men: 3.0 [SD 2.97]; women: 4.4 [SD 3.69]). Among the 32 patients, 16 (50%) had lung metastases, 4 (12.5%) had bone metastases, 4 (12.5%) had liver metastases, 3 (9.4%) had adrenal gland metastases, and 12 (37.5%) had metastases at other locations.

During the study, each patient received a median of 6.5 tivozanib treatment cycles (range 1–35) and the median time on treatment was 5.7 months (range 0.4–32.7). Tivozanib was administered at a starting dose of 1340 micrograms in 26 patients (81.3%) and at 890 micrograms in six patients (18.8%); however, the reason for this was not documented. Pre- or co-medication was required by nine patients (28.1%) and reasons for use included emesis (n = 3 [9.4%]), infection (n = 2 [6.3%]), skin reactions (n = 1 [3.1%]), allergic reactions (n = 1 [3.1%]) and ‘other reasons’ (n = 8 [25.0%]); some patients required pre- or co-medication for more than one reason. Prednisolone was administered in one patient (3.1%) and another active ingredient was administered in four patients (12.5%).

### 3.2. Safety Outcomes

Treatment-related adverse events were experienced by 25 patients (78.1%). Grade 1–2 events included diarrhea (n = 6 [18.8%]), nausea (n = 7 [21.9%]), stomatitis (n = 2 [6.3%]), peripheral neuropathy (n = 1 [3.1%]), emesis (n = 1 [3.1%]), and alopecia (n = 1 [3.1%]) (Table 2). Adverse events that were not graded and were experienced by ≥5% of patients included hypotension/hypertension (n = 5 [15.6%]), skin changes (n = 3 [9.4%]), neurologic disorders (n = 2 [6.3%]), paresthesia (n = 2 [6.3%]), fatigue (n = 2 [6.3%]), and ‘other’ (n = 22 [68.8%]). Grade 3–4 adverse events included cardiac dysfunction (n = 2 [6.3%]), diarrhea (n = 2 [6.3%]), ataxia (n = 1 [3.1%]), aphonia (n = 1 [3.1%]), pericardial effusion (n = 1 [3.1%]), and ‘other’ (n = 5 [15.6%]).

Treatment interruption and dose reduction were required in eight patients (25.0%) each. Seven patients (21.9%) required a dose reduction due to adverse events and one patient (3.1%) requested a dose reduction. Nine patients (28.1%) required hospitalization; however, the reasons were not documented. Early treatment discontinuations occurred in seven patients (21.9%) and were due to patient request (n = 2 [6.3%]), adverse events (n = 1 [3.1%]) and ‘other reasons’ (n = 4 [12.5%]).

### 3.3. Patient Reported Outcomes

**Figure 2:**
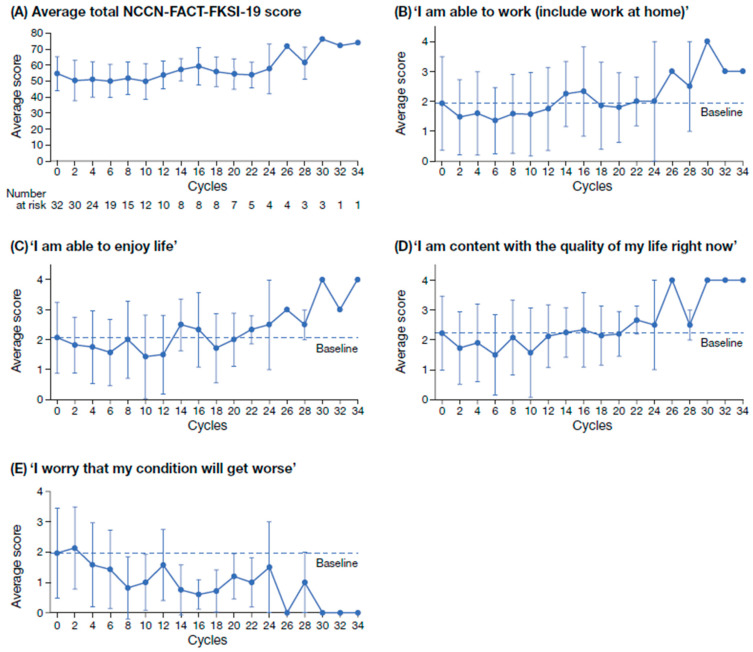
Average total NCCN-FACT-FKSI-19 score at alternating treatment cycles (**A**) and scores on the QoL sub-scales ‘I am able to work (include work at home)’ (**B**); ‘I am able to enjoy life’ (**C**); ‘I am content with the quality of my life right now’ (**D**); ‘I worry that my condition will get worse’ (**E**). Scores on the sub-scales in B–E range from 0 (not at all) to 4 (very much). NCCN-FACT-FKSI-19, National Comprehensive Cancer Network Functional Assessment for Cancer Therapy—Kidney Symptom Index; QoL, quality of life.

In total, 149 NCCN-FACT-FKSI-19 questionnaires were completed by patients treated with tivozanib. Per cycle, a minimum of 1 questionnaire and a maximum of 22 questionnaires were completed. The overall completion rate of the NCCN-FACT-FKSI-19 was 76.8% (Figure 2 and Figure S1). At baseline, questionnaire completion rate per patient was 100% (n = 32); however, the number of patients completing the questionnaire decreased over the course of the study. For all 19 QoL items evaluated using the PRO questionnaire NCCN-FACT-FKSI-19, there was no decrease in the mean score with increasing cycles (Figure 2A). For the sub-scales ‘I am able to work (include work at home)’, ‘I am able to enjoy life’, and ‘I am content with the quality of my life right now’, mean NCCN-FACT-FKSI-19 scores generally increased from cycle 2 to cycle 34 (Figure 2B–D). For the sub-scale ‘I worry that my condition will get worse’, the mean NCCN-FACT-FKSI-19 score generally decreased from cycle 2 to cycle 34 (Figure 2E). For the remaining 15 NCCN-FACT-FKSI-19 scores, the same trend of improvement over time was observed (Figure S2).

For most of the symptomatic toxicities evaluated with the PRO-CTCAE questionnaire, the frequency and severity of the symptoms, as well as their interference with normal daily activities, decreased from baseline to cycle 34. While the majority of symptoms appeared to resolve prior to cycle 34 in most patients, one patient reported the severity of dry skin, itching and hand–foot syndrome worsened from baseline to cycle 34; dry skin was reported to be ‘fairly’, and both itching and hand–foot syndrome were reported to be ‘moderate’ at study completion. As with the NCCN-FACT-FKSI-19, the proportion of patients completing the PRO-CTCAE questionnaire decreased as the study continued.

### 3.4. Effectiveness Outcomes

**Figure 3:**
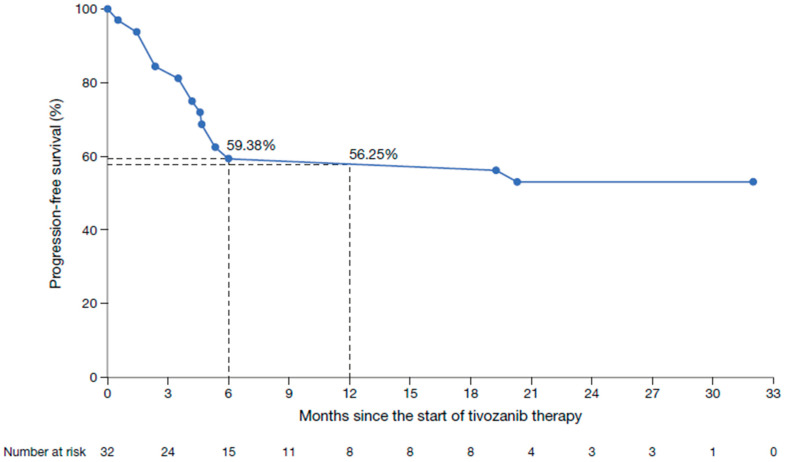
Kaplan–Meier plot of clinical progression-free survival in patients treated with tivozanib in a first-line setting.

Clinical response rate was reported in 15 patients (46.9%), with six patients (18.8%) achieving a complete response and nine patients (28.1%) a partial response (Table 3). Stable disease was reported in four patients (12.5%), giving a clinical disease control rate of 59.4%. Progressive disease in patients without prior response was observed in nine patients (28.1%). The clinical progression-free survival rates were 59.4% after 6 months and 56.3% after 12 months of tivozanib treatment (Figure 3); median clinical progression-free survival was not reached. Treatment with tivozanib was discontinued in 11 patients (34.4%); 9 (28.1%) discontinued due to disease progression and two patients (6.3%) died while receiving study treatment.

## 4. Discussion

The prospective, non-interventional T-Rex study evaluated the safety, effectiveness and impact on QoL of tivozanib in patients with advanced or mRCC treated in post-approval clinical practice across Germany. Our results demonstrate that first-line treatment with tivozanib was generally well tolerated, with no new safety signals observed during the study. Tivozanib also demonstrated efficacy and maintained QoL in our study population.

As expected, the baseline characteristics of the patient population included in this study differ from those of the population included in the pivotal Phase III clinical trial, TIVO-1, which investigated the efficacy and safety of tivozanib in comparison with sorafenib in patients with mRCC [10]. Compared with patients included in TIVO-1, the population enrolled in T-Rex had a higher median age (77.5 years versus 59.0 years) and included a greater proportion of patients with a favorable ECOG PS of 0 (65.6% versus 49.3%). However, while TIVO-1 only included patients with an ECOG PS ≤ 1, the T-Rex study enrolled two patients (6.3%) with an unfavorable ECOG PS of 2. Advanced age may be associated with patient frailty and vulnerability in the clinical setting [46], and recent evidence suggests that frailty can affect >50% of older patients with cancer [47]. The median patient age in T-Rex (77.5 years) was also higher compared with that of patient populations included in other Phase III studies in advanced or mRCC, which included patient populations with a median age of approximately 60 years [12,13,24,25]. In T-Rex, 81.3% of the population were aged >65 years, suggesting that a large proportion of this population would be at risk of frailty. This observation is further supported by the high number of patients with comorbidities, as indicated by the high mean CCI score. Although such data are lacking for pivotal TKI trials such as TIVO-1, they emphasise the influence of competing risks and comorbidities in patients in routine clinical practice.

In this post-approval study, first-line treatment with tivozanib resulted in a clinical response rate of 46.9%, confirming its effectiveness in everyday clinical practice, including in elderly patients, who, as discussed above, have a high probability of being frail. Clinical progression-free survival rates at 6 and 12 months were comparable between the T-Rex study and the cohort receiving tivozanib in TIVO-1, despite T-Rex including a patient population that was older than that in TIVO-1. Consistent with our findings, another post-approval study of tivozanib reported a significant improvement in PFS, with greater benefits seen in patients with metastatic RCC treated in the first-line setting [48]. The safety profile of tivozanib in T-Rex was similar to that reported previously [10,27,49,50], despite the advanced age and associated comorbidities of the study population, as measured by the CCI. Overall, adverse events were experienced by 78.1% of patients, primarily diarrhea, nausea and hypertension/hypotension, and no new safety signals were observed. This incidence is slightly lower than that reported in TIVO-1, where adverse events occurred in 93.6% of patients, with hypertension, diarrhea and dysphonia being the most commonly observed [10]. Furthermore, the T-Rex study reported low rates of grade 3–4 adverse events, such as cardiac dysfunction (6.3%) and diarrhea (6.3%). These rates were comparable to, or lower than, those observed in the TIVO-1 trial, which reported grade 3–4 hypertension in 27% and diarrhea in 2% of patients [10]. In another post-approval study of first-line tivozanib use in patients with RCC, the tolerability of tivozanib appears favorable vs. other TKIs in this setting, but the authors noted that adverse events are likely to be under-recorded in real-world studies [51]. Additionally, outcomes cannot be directly compared between studies due to differences in study design and patient populations. It is also generally accepted that real-world studies are less accurate than clinical trials in the reporting of adverse events [32]. In total, 9 patients experienced 12 serious adverse events over the course of the study, further demonstrating the favorable tolerability profile of tivozanib in our patient population. The safety of other TKIs, including cabozantinib, sunitinib and pazopanib, has been evaluated in other real-world studies, which reported comparable findings to the T-Rex study [52,53]. In these studies, adverse events were observed in 91% of patients [52], while grade ≥ 3 events occurred in 22–33% [52,53].

The T-Rex study also evaluated PRO, which are an important tool for the assessment of the patients’ QoL and are valuable in guiding treatment decisions and improving patient well-being [39,41,54]. Patient scores generally improved for all items included in the NCCN-FACT-FKSI-19 questionnaire, suggesting that overall patient QoL was maintained throughout the duration of the study [9]. For the sub-scales ‘I am able to work (include work at home)’, ‘I am able to enjoy life’, and ‘I am content with the quality of my life right now’, scores increased over the course of the study. Patients also reported improvements in their overall condition with continued tivozanib use, with scores generally decreasing for the sub-score ‘I worry that my condition will get worse’. The absence of a decline in these PRO items potentially indicates that therapy management allowed patients to adapt to treatment toxicities and the burden of treatment. However, drop-outs, missing PRO assessments and a small sample size limit the interpretation of the data. According to patient responses on the PRO-CTCAE questionnaire, the frequency, severity, and interference with normal daily activities of most of the evaluated symptomatic toxicities decreased from baseline to study completion. However, the evaluation of PRO was challenging, as the number of patients completing the questionnaires reduced throughout the course of the study, introducing selection bias to the results.

There are several important limitations to consider regarding this study. Firstly, the small sample size included in T-Rex makes it challenging to interpret the results and draw conclusions from the study. Secondly, the lack of a comparator further limits the interpretability of these findings, which is, however, inherent to non-interventional studies. In addition, the absence of standardized imaging assessments for tumor evaluation may have introduced variations in response assessment across treatment centers. Finally, it is possible that adverse events were underreported. Despite these limitations, the present study provides meaningful evidence on the clinical effectiveness, safety profile and impact on QoL of first-line treatment with tivozanib in patients with advanced and mRCC, complementing data from other global clinical trials.

## 5. Conclusions

First-line treatment with tivozanib demonstrated relevant clinical activity and favorable tolerability in patients with advanced or mRCC in routine clinical practice in Germany, including elderly and potentially frail patients. The results of this study support the use of tivozanib for the first-line treatment of patients with advanced or mRCC suitable for single-agent TKI treatment.

## Figures and Tables

**Table 1:** Baseline demographic and clinical characteristics.

Characteristic	N = 32
Male gender, n (%)	19 (59.4)
Median age, years (range)	77.5 (61.0–89.0)
≤65 years, n (%)	6 (18.8)
66–75 years, n (%)	9 (28.1)
>75 years, n (%)	17 (53.1)
Histology at diagnosis, n (%)	
Clear cell RCC	25 (78.1)
Clear cell papillary RCC	1 (3.1)
Papillary RCC, types I + II	2 (6.3)
Chromophobic RCC	2 (6.3)
RCC (unclassifiable)	2 (6.3)
Mean Charlson Comorbidity Index excluding points for age and solid tumors (range)	3.5 (0.0–11.0)
ECOG PS, n (%)	
0	21 (65.6)
1	9 (28.1)
2	2 (6.3)
IMDC, n (%)	
Favorable	7 (21.8)
Intermediate	22 (68.8)
Poor	3 (9.4)
Fraction of patients with nephrectomy, n (%)	24 (75.0)
Patients with metastases (M1) *	26 (81.3)
Lung metastases, n (%)	16 (50)
Bone metastases, n (%)	4 (12.5)
Liver metastases, n (%)	4 (12.5)
Adrenal gland metastases, n (%)	3 (9.4)
Other locations, n (%)	12 (37.5)
Mean number of tivozanib cycles received (range)	10.6 (1–35)
Median	6.5
Median treatment duration, months (range)	5.7 (0.4–32.7)

ECOG, Eastern Cooperative Oncology Group; IMDC, International mRCC Database Consortium; PS, performance status; RCC, renal cell carcinoma. * Documented for 26 patients (6 unclear).

**Table 2:** Incidence of treatment-emergent adverse events of any grade occurring during the study and up to 30 days following the last dose of tivozanib.

Adverse Event	All Grades	Not Graded	Grade 1–2	Grade 3–4
Total adverse events, n (%)				
Diarrhea	8 (25.0)	0 (0.0)	6 (18.8)	2 (6.3)
Nausea	7 (21.9)	0 (0.0)	7 (21.9)	0 (0.0)
Hypotension/Hypertension	5 (15.6)	5 (15.6)	0 (0.0)	0 (0.0)
Cardiac dysfunction	3 (9.4)	1 (3.1)	0 (0.0)	2 (6.3)
Skin changes	3 (9.4)	3 (9.4)	0 (0.0)	0 (0.0)
Stomatitis	2 (6.3)	0 (0.0)	2 (6.3)	0 (0.0)
Ataxia	2 (6.3)	1 (3.1)	0 (0.0)	1 (3.1)
Paresthesia	2 (6.3)	2 (6.3)	0 (0.0)	0 (0.0)
Fatigue	2 (6.3)	2 (6.3)	0 (0.0)	0 (0.0)
Neurologic disorders	2 (6.3)	2 (6.3)	0 (0.0)	0 (0.0)
Emesis	1 (3.1)	0 (0.0)	1 (3.1)	0 (0.0)
Alopecia	1 (3.1)	0 (0.0)	1 (3.1)	0 (0.0)
Peripheral neuropathy	1 (3.1)	0 (0.0)	1 (3.1)	0 (0.0)
Aphonia	1 (3.1)	0 (0.0)	0 (0.0)	1 (3.1)
Taste disturbance	1 (3.1)	1 (3.1)	0 (0.0)	0 (0.0)
Pericardial effusion	1 (3.1)	0 (0.0)	0 (0.0)	1 (3.1)
Pruritus	1 (3.1)	1 (3.1)	0 (0.0)	0 (0.0)
Pain	1 (3.1)	1 (3.1)	0 (0.0)	0 (0.0)
Other	22 (68.8)	17 (53.1)	0 (0.0)	5 (15.6)

**Table 3:** Clinical tumor response following first-line treatment with tivozanib.

Response Category, n (%)	N = 32 *
Clinical response rate	15 (46.9)
CR	6 (18.8)
PR	9 (28.1)
SD	4 (12.5)
PD	9 (28.1)
Unclear	4 (12.5)
DCR	19 (59.4)

* Includes four patients with unknown response (for one of these patients, documented as “unclear”, “death” was reported). CR, complete response; DCR, disease control rate; PD, progressive disease; PR, partial response; SD, stable disease.

## Data Availability

The original data presented in the study are openly available at the Federal Institute for Drugs and Medical Devices at https://awbdb.bfarm.de/ords/r/awb/awb/anzeigedetails?p2_anzeige_id=40BB22D754093D83E063140810AC1B5D&clear=2 (accessed on 1 November 2025) or ID 7296.

## Supplementary Materials

The following supporting information can be downloaded at: https://www.mdpi.com/article/10.3390/cancers17243910/s1, Figure S1: Proportion of patients completing the NCCN-FACT-FKSI-19 questionnaire at alternate treatment cycles; Figure S2: NCCN-FACT-FKSI-19 score on the QoL sub-scales.
