# Supplementary material for: First-Line Treatment with Tivozanib for Metastatic Renal Cell Carcinoma in Real-World Settings Across Germany: Results of the Prospective, Non-Interventional, Post-Approval Study T-Rex
Source: Cancers (Basel). 2025 Dec 7;17(24):3910. doi: 10.3390/cancers17243910 (PMC12730253; doi:10.3390/cancers17243910)
Supplement: Supplementary file 1 [file cancers-17-03910-s001.zip › cancers-3961019-supplementary.pdf]

Figure S1. Proportion of patients completing the NCCN-FACT-FKSI-19 questionnaire at alternate treatment cycles. NCCN-FACT-FKSI-19, National Comprehensive Cancer Network Functional Assessment for Cancer Therapy – Kidney Symptom Index

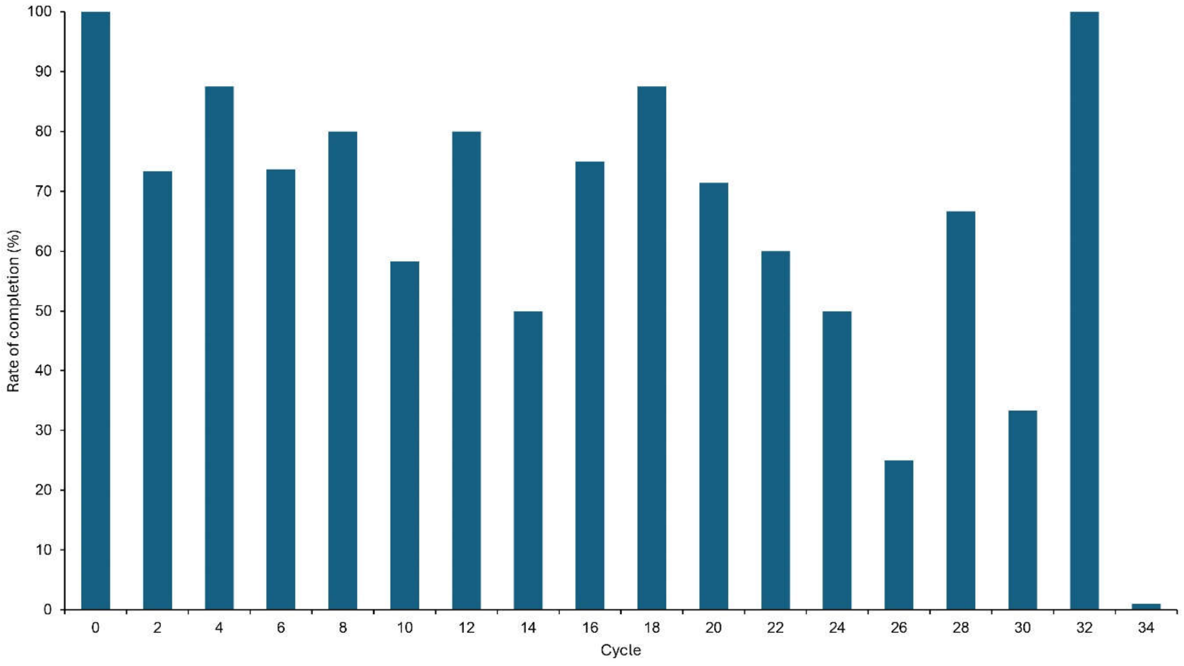

Figure S2. NCCN-FACT-FKSI-19 score on the QoL sub-scales: (A) 'I have a lack of energy'; (B) 'I have pain'; (C) 'I am losing weight'; (D) 'I feel fatigued'; (E) 'I have been short of breath'; (F) 'I am bothered by fevers (episodes of high body temperature)'; (G) 'I have bone pain'; (H) I have been coughing'; (I) 'I feel weak all over'; (J) 'I have had blood in my urine'; (K) 'I have a good appetite'; (L) 'I am sleeping well'; (M) 'I have nausea' (N) 'I have diarrhea (diarrhoea)' (O) 'I am bothered by side effects of treatment'. Scores on the sub-scales range from 0 (not at all) to 4 (very much). NCCN-FACT-FKSI-19, National Comprehensive Cancer Network Functional Assessment for Cancer Therapy – Kidney Symptom Index; QoL, quality of life.

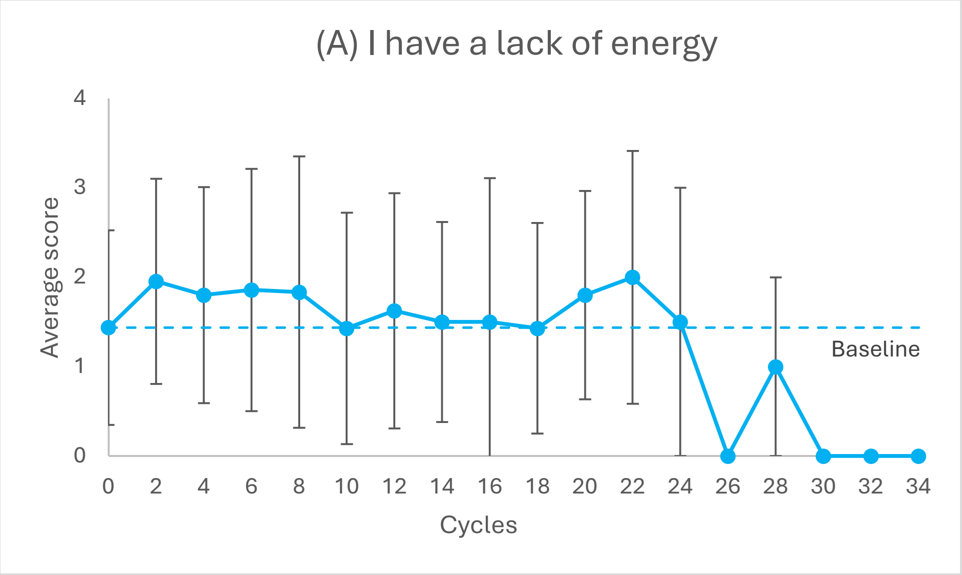

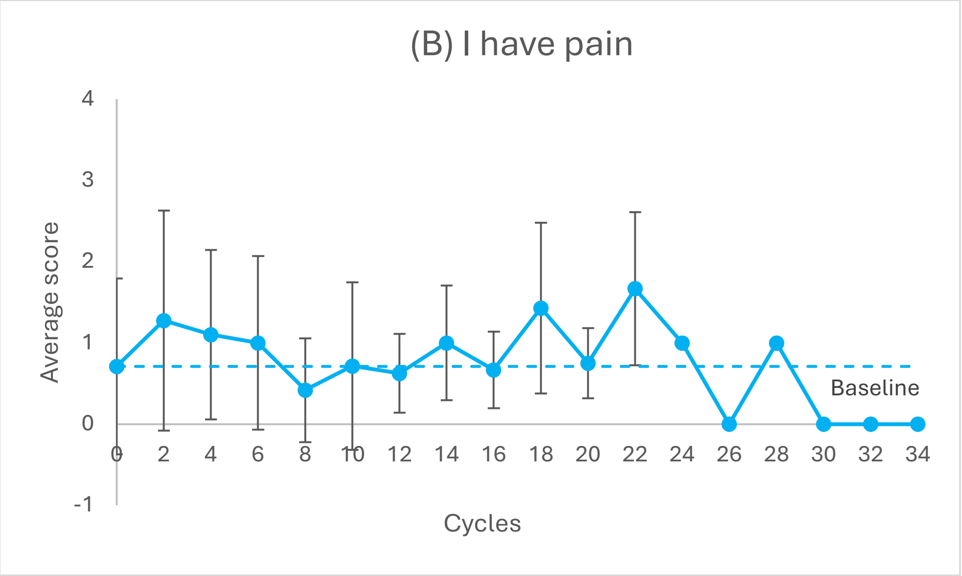

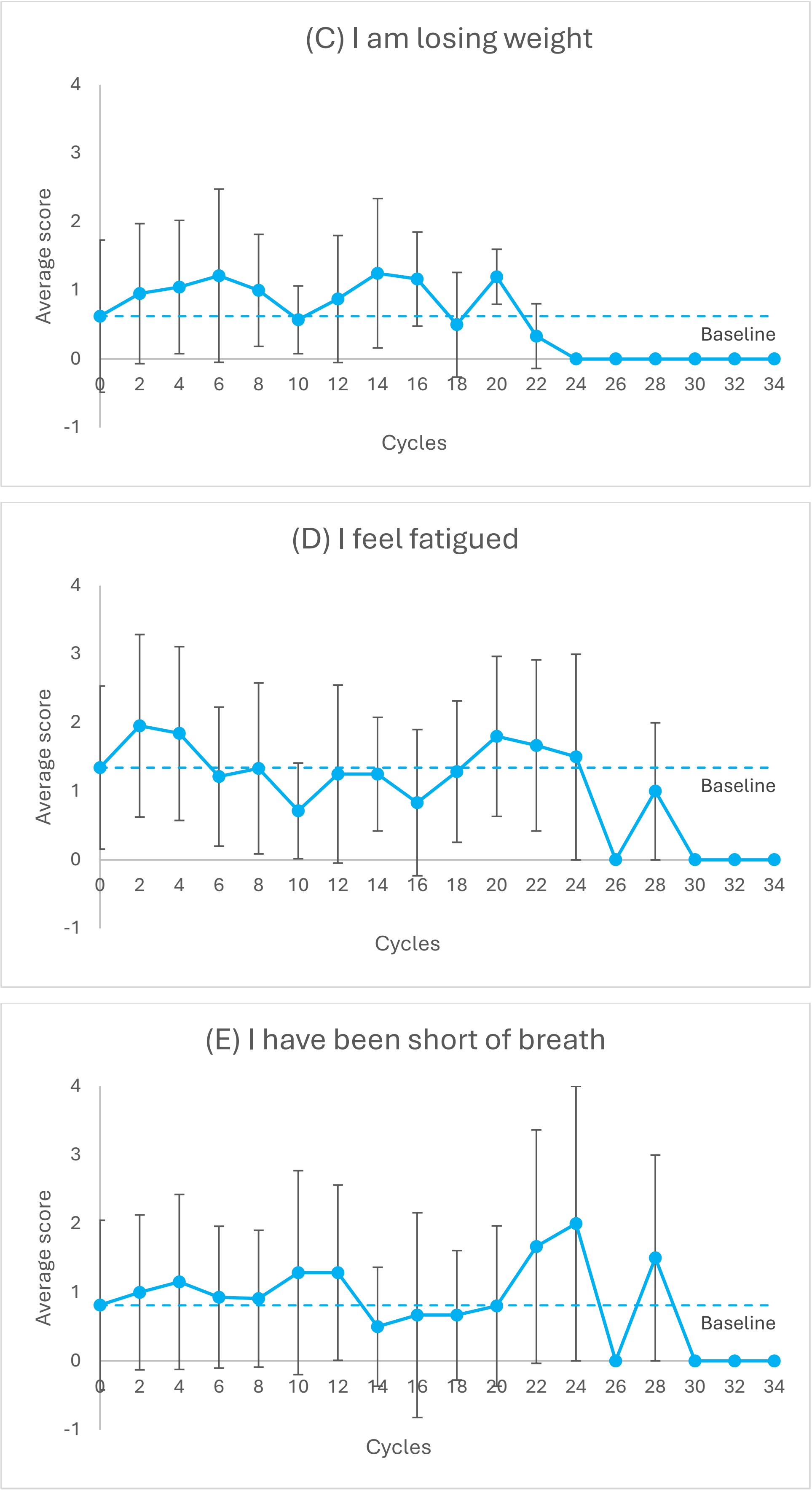
(C) I am losing weight
Average score
4
3
2
1
0
-1
0
2
4
6
8
10
12
14
16
18
20
22
24
26
28
30
32
34
Baseline
Cycles
(D) I feel fatigued
Average score
4
3
2
1
0
-1
0
2
4
6
8
10
12
14
16
18
20
22
24
26
28
30
32
34
Baseline
Cycles
(E) I have been short of breath
Average score
4
3
2
1
0
-1
0
2
4
6
8
10
12
14
16
18
20
22
24
26
28
30
32
34
Baseline
Cycles

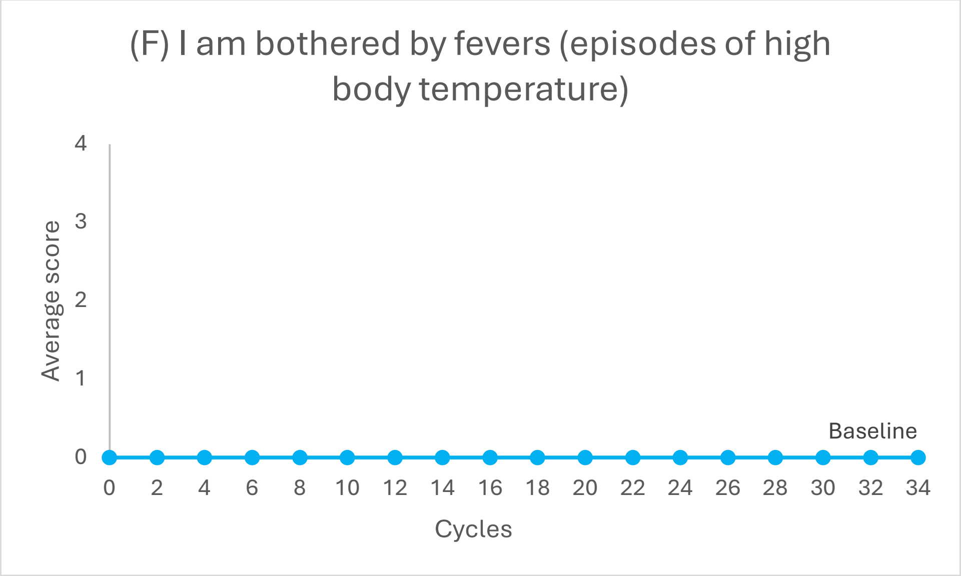

(F) I am bothered by fevers (episodes of high body temperature)
Average score
Baseline
Cycles

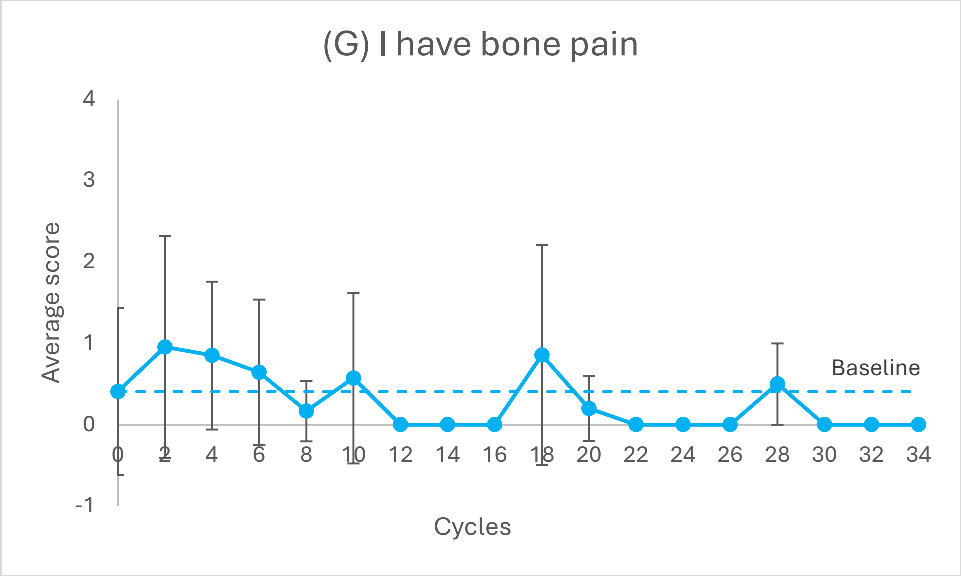

(G) I have bone pain
Average score
Baseline
Cycles

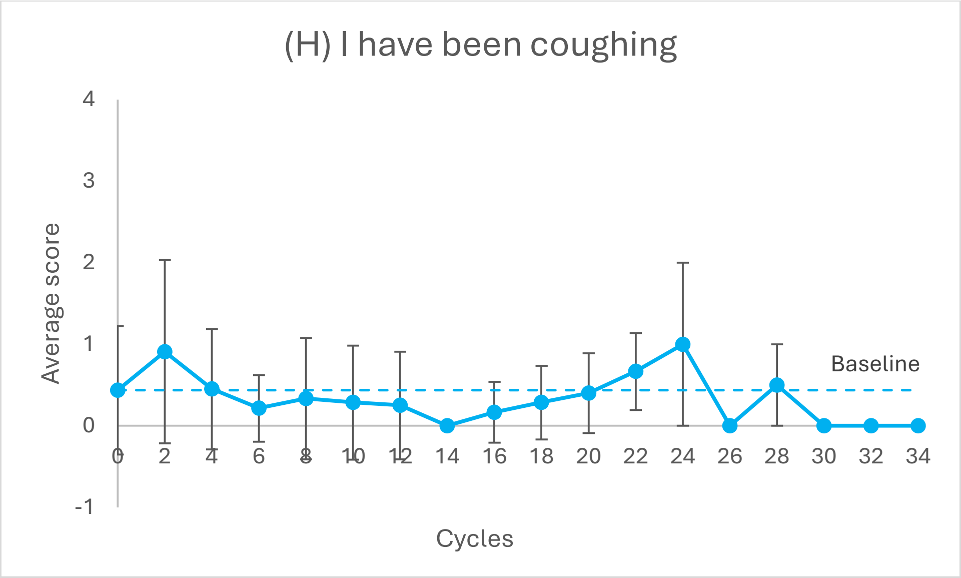

(H) I have been coughing
Average score
Baseline
Cycles

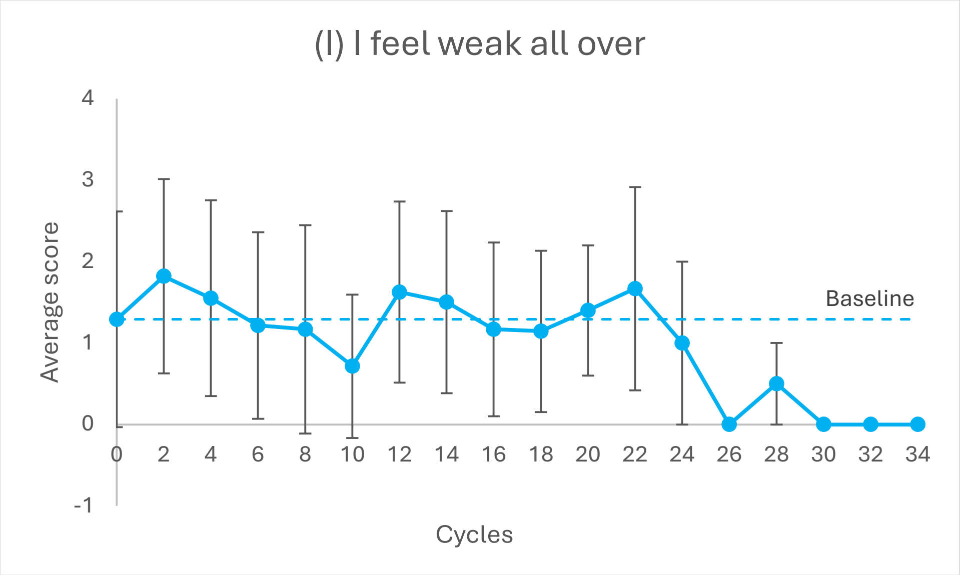
(I) I feel weak all over
Average score
Cycles
Baseline
4
3
2
1
0
-1
0 2 4 6 8 10 12 14 16 18 20 22 24 26 28 30 32 34

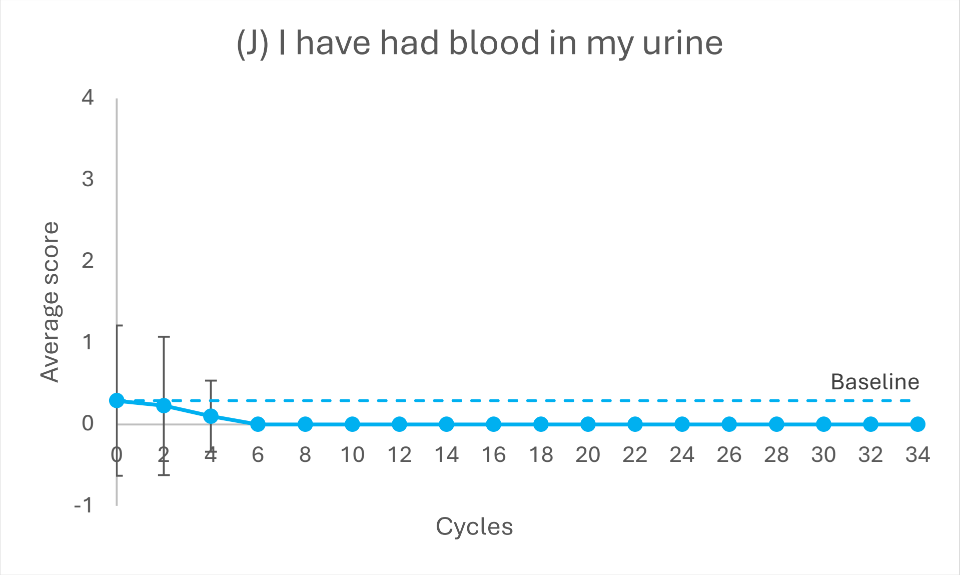
(J) I have had blood in my urine
Average score
Cycles
Baseline
4
3
2
1
0
-1
0 2 4 6 8 10 12 14 16 18 20 22 24 26 28 30 32 34

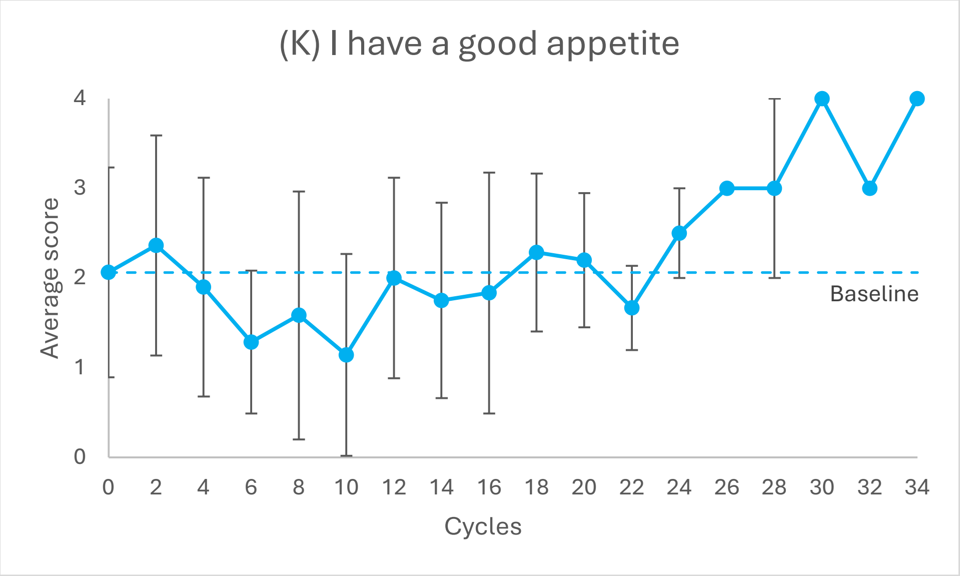
(K) I have a good appetite
Average score
Cycles
Baseline
4
3
2
1
0
0 2 4 6 8 10 12 14 16 18 20 22 24 26 28 30 32 34

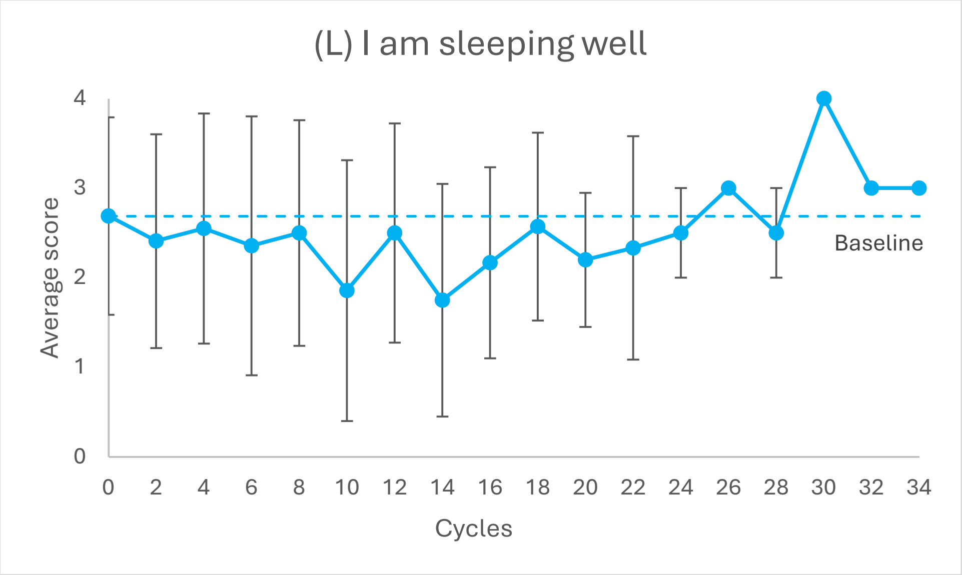
(L) I am sleeping well
Average score
4
3
2
1
0
0 2 4 6 8 10 12 14 16 18 20 22 24 26 28 30 32 34
Cycles
Baseline

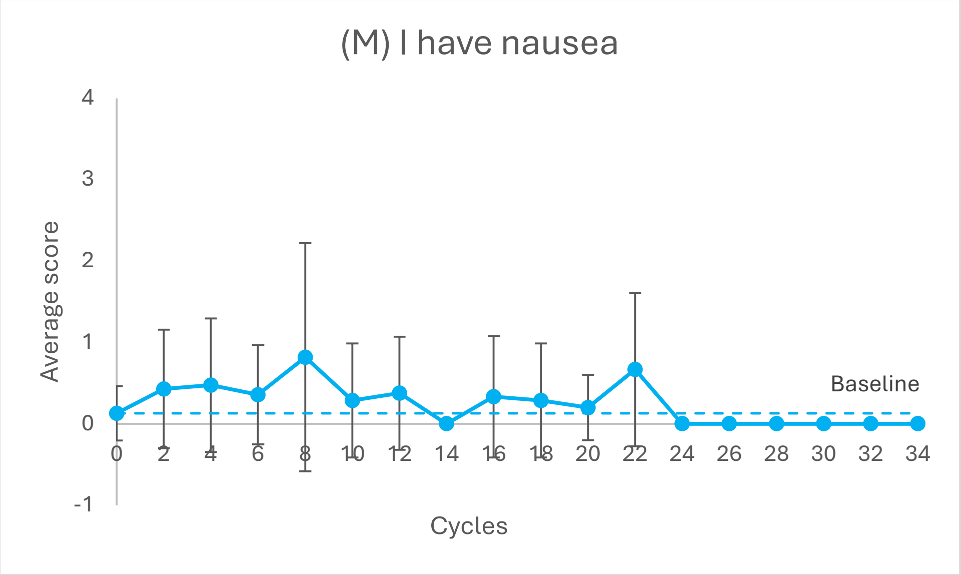
(M) I have nausea
Average score
4
3
2
1
0
-1
0 2 4 6 8 10 12 14 16 18 20 22 24 26 28 30 32 34
Cycles
Baseline

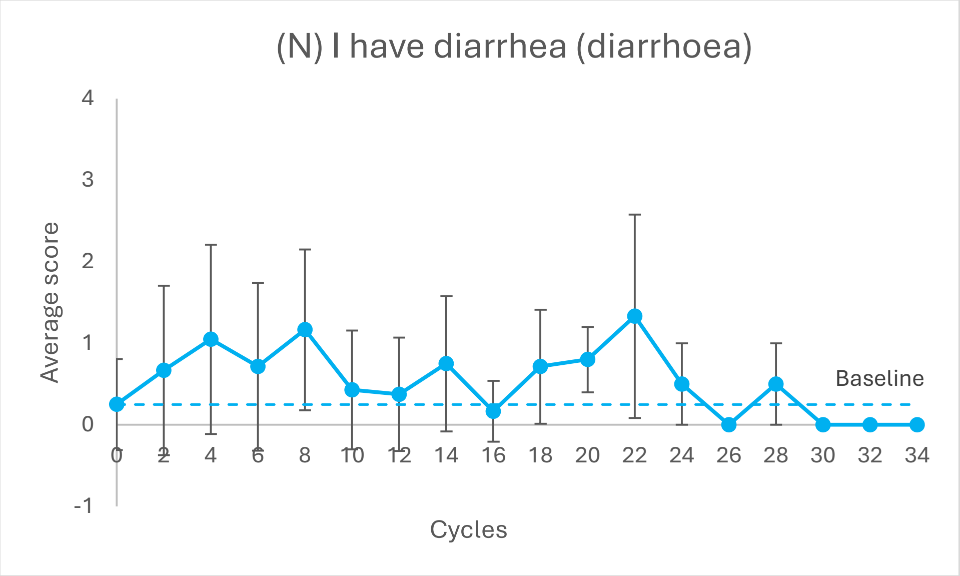
(N) I have diarrhea (diarrhoea)
Average score
4
3
2
1
0
-1
0 2 4 6 8 10 12 14 16 18 20 22 24 26 28 30 32 34
Cycles
Baseline

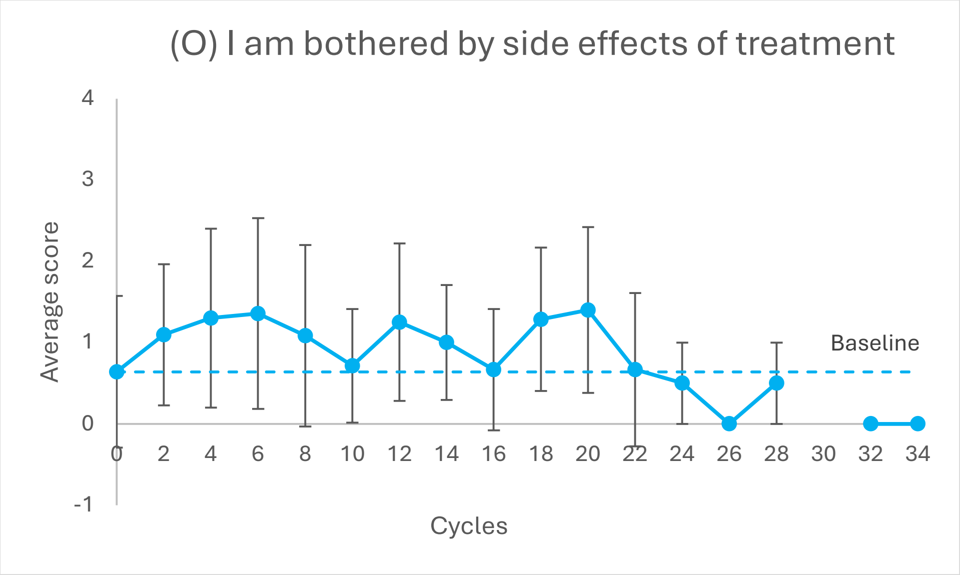

(O) I am bothered by side effects of treatment
Average score
4
3
2
1
0
-1
Baseline
0
2
4
6
8
10
12
14
16
18
20
22
24
26
28
30
32
34
Cycles
